# Ongoing outbreak of hepatitis A associated with sexual transmission among men who have sex with men, Portugal, October 2023 to April 2024

**DOI:** 10.2807/1560-7917.ES.2024.29.21.2400272

**Published:** 2024-05-23

**Authors:** Ebba Rosendal, Sebastian von Schreeb, Alexandre Gomes, Sara Lino, Berta Grau-Pujol, Sara Magalhães, Vasco Ricoca Peixoto, Carla Roque, Joana Moreno, Fernando Maltez, Fernando Almeida, Rita Sá Machado, Rui Tato Marinho, Paula Vasconcelos, Rita de Sousa, João Vieira Martins

**Affiliations:** 1 ECDC Fellowship Programme, Public Health Microbiology path (EUPHEM), European Centre for Disease Prevention and Control (ECDC), Stockholm, Sweden; 2 Infectious Diseases Department, National Institute of Health Doctor Ricardo Jorge, Lisbon, Portugal; 3 ECDC Fellowship Programme, Field Epidemiology path (EPIET), European Centre for Disease Prevention and Control (ECDC), Stockholm, Sweden; 4 Center for Public Health Emergencies, Directorate-General of Health, Lisbon, Portugal; 5 Directorate of Information and Analysis, Directorate-General of Health, Lisbon, Portugal; 6 National Programmes for Tuberculosis, Viral Hepatitis, STI and HIV, Directorate-General of Health, Lisbon, Portugal; 7 Infectious Diseases Department, Hospital de Curry Cabral; Unidade Local de Saúde de São José, Lisbon, Portugal; 8 National Institute of Health Doctor Ricardo Jorge, Lisbon, Portugal; 9 Directorate-General of Health, Lisbon, Portugal; 10 Medical School of Lisbon, Universidade de Lisboa; Local Unit of Health Santa Maria; National Programme for Viral Hepatitis, Directorate-General of Health; Lisbon, Portugal; * These authors contributed equally to this work and share first authorship.; ** These authors contributed equally to this work and share last authorship.

**Keywords:** Hepatitis A, Hepatitis A virus, outbreak, MSM, sexual transmission, genotyping, Portugal

## Abstract

An outbreak of hepatitis A is ongoing in Portugal, with 71 confirmed cases from 7 October 2023 to 24 April 2024. Most cases are male, aged 18–44 years, with many identifying as men who have sex with men (MSM) and reported as suspected sexual transmission. Phylogenetic analysis identified the subgenotype IA, VRD 521–2016 strain, last observed in an MSM-associated multi-country outbreak in 2016 to 2018. We wish to alert colleagues in other countries to investigate potential similar spread.

Portugal is classified as a very low-endemicity country for hepatitis A [1,2]. Since 2007, 10–82 cases have been reported annually, with the exception of a global multi-country outbreak in 2016 to 2018 that largely affected men who have sex with men (MSM) [3,4]. In early 2024, an increase in hepatitis A cases was detected, and preliminary investigations suggested sexual transmission. A task force was established to investigate the outbreak. Here, we describe the findings of the investigation and discuss the public health response, aiming to raise awareness and provide guidance for managing similar outbreaks, especially considering the upcoming summer season with its social gatherings and events.

## Outbreak detection and case definition

Through the national surveillance system, an unusually high number of hepatitis A cases was reported with symptom onset in mid-January 2024 (Figure), between five and six cases per week compared with an average of 0.5 cases per week during the previous 4 years. Concurrently, clinical reports and early investigations suggested that many cases were MSM, while also raising concern of sexual transmission. Molecular surveillance identified several cases with the same subgenotype IA HAV strain (VRD 521–2016), indicating a linked outbreak within this community. As the high number of cases persisted in the following week, an alert was issued on the European Centre for Disease Prevention and Control (ECDC) EpiPulse platform on 28 February 2024, followed by a public health warning from the Directorate-General of Health (DGS) on 12 March 2024.

**Figure:**
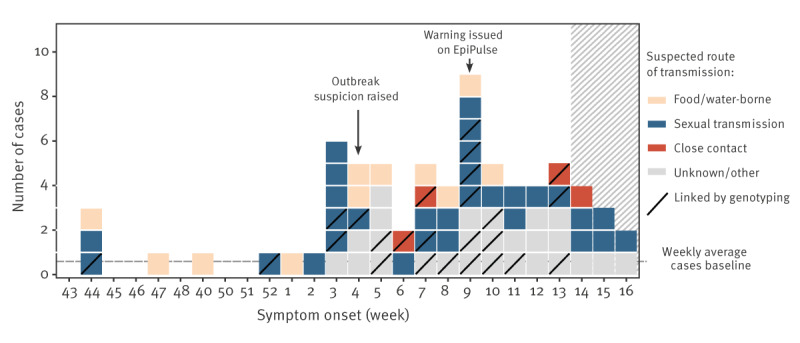
Confirmed hepatitis A cases by date of symptom onset, Portugal, 7 Oct 2023–24 April 2024 (n = 71)

Coloured by reported suspected transmission route and molecular typing results. Dotted line shows average weekly cases during baseline (1 Jan 2020–6 Oct 2023). The shaded area shows the upper quartile of time from symptom onset to case confirmation, illustrating the period of uncertainty in reported case numbers. ‘Close contact’ means reported as suspected person-to-person transmission excluding sexual transmission.

For the outbreak investigation, confirmed cases were defined as any person with clinical symptoms consistent with acute hepatitis A and a laboratory-confirmed HAV infection according to the European Union case definition for surveillance [5], with onset of symptoms since 7 October 2023, i.e. 100 days before the surge in cases (two times the maximum incubation period).

## Epidemiological investigation

We extracted epidemiological data from the national surveillance system (SINAVE). It includes nationwide notifications reported by clinicians and laboratories and epidemiological investigation data from local public health doctors, verified at regional and national levels.

To better understand the specific characteristics of this outbreak, we employed a case–case design comparing the outbreak period (Oct 2023–April 2024; n = 71) with hepatitis A cases from the baseline period before the outbreak (Jan 2020–Oct 2023; n = 101) (Table). Furthermore, we conducted a sub-analysis comparing against the baseline only outbreak cases linked by genotyping (strain IA, VRD 521–2016). The incidence rate was six times higher in the outbreak period compared with the baseline (1.8 vs 0.3 cases per 100,000 people and year). Notably, no seasonal pattern of hepatitis A cases were observed during the baseline period (data not shown). Compared with the baseline, outbreak cases were more likely to be men (odds ratio (OR) = 4.0; 95% confidence interval (CI): 2.0–9.0), aged 18–44 years (OR = 9.0; 95% CI: 3.2–32.6) and reported from the Lisbon region (OR = 4.0; 95% CI: 1.8–9.5). 

**Table:** Characteristics of confirmed hepatitis A cases, Portugal, outbreak period (7 Oct 2023–24 April 2024) vs baseline period (1 Jan 2020–6 Oct 2023), and outbreak cases linked by genotyping vs baseline period

	Baseline	%	Outbreak total	%	OR^a,b^	95% CI	Outbreak cases linked by genotyping^c^	%	OR^a,b^	CI
**Total**	**101**	**100**	**71**	**100**	** NA**		**25**	**100**	** NA**	
**Age category (years)**										
0–17	23	23	3	4.2	Reference		0	0	Reference	
18–44	37	37	58	82	9.0***	3.2–32.6	25	100	15.5**	3.0–286.9
45–64	25	25	7	9.9	1.6	0.4–6.8	0	0	NA	
≥ 65	16	16	3	4.2	1.1	0.2–5.56	0	0	NA	
**Sex (collected as binary variable)**										
Female	43	43	11	15	Reference		3	12	Reference	
Male	58	57	60	85	4.0***	2.0–9.0	22	88	5.4**	1.7–24.0
**MSM^d^**										
No	2	3.4	0	0	Reference		0	0	Reference	
Yes	1	1.7	25	42	19.6**	3.9–358.0	9	41	20.2**	3.4–390.0
Unknown	55	95	35	58	NA		13	59	NA	
**HIV^d^**										
No	32	32	56	79	Reference		17	68	Reference	
Yes	0	0	14	20	8.0*	1.5–148.3	8	32	15.1*	2.5–291.6
Unknown	69	68	1	1.4	NA		0	0	NA	
**Suspected mode of transmission**										
Alimentary/water	22	22	11	15	Reference		0	0	Reference	
Close contact	9	8.9	4	5.6	0.8	0.2–3.1	3	12	7.3	0.8–159.8
Sexual transmission	3	3.0	30	42	18.3***	5.2–88.3	11	44	80.7***	10.7–1,793.0
Other/unknown	67	66	26	37	0.7	0.3–1.7	11	44	3.6	0.7–67.9
**Country of birth**										
Portugal	61	60	34	48	Reference		11	44	Reference	
Brazil	4	4.0	24	34	10.8***	3.8–39.0	13	52	18.0***	5.3–74.1
Other	34	34	13	18	0.7	0.3–1.5	1	4.0	0.2	0.0–0.9
Unknown	2	2.0	0	0	NA		0	0	NA	
**Travel history^e^**										
No travel	58	57	46	65	Reference		19	76	Reference	
Brazil	0	0	4	5.6	5.0	0.7–100.5	1	4.0	3.1	0.1–79.7
Other destinations	12	12	16	23	1.7	0.7–4.0	3	12	0.8	0.1–2.7
Unknown	31	31	5	7.0	NA		2	8.0	NA	
**Region**										
Norte	34	34	10	14	Reference		1	4.0	Reference	
Centro	9	8.9	4	5.6	1.5	0.4–5.8	0	0		
LVT	43	43	51	72	4.0***	1.8–9.5	23	92	18.2**	3.6–333.5
Alentejo	4	4.0	2	2.8	1.7	0.2–10.1	0	0		
Algarve	8	7.9	4	5.6	1.7	0.4–6.7	1	4.0	4.3	0.2–115.6
Açores	2	2.0	0	0	NA		0	0	NA	
Madeira	1	1.0	0	0	NA		0	0	NA	
**Education level based on occupation**										
Basic	25	25	19	27	Reference		7	28	Reference	
Intermediate	5	5.0	5	7.0	1.3	0.3–5.4	2	8.0	1.4	0.18–8.4
Higher	7	6.9	8	11	1.5	0.5–5.0	5	20	2.6	0.6–10.8
Unknown	64	63	39	55	NA		11	44	NA	
**Hospitalised**										
No	48	48	25	35	Reference		10	40	Reference	
Yes	51	50	45	63	1.7	0.9–3.2	15	60	1.4	0.6–3.5
Unknown	2	2.0	1	1.4	NA		0	0	NA	

CI: confidence interval; HIV: human immunodeficiency virus infection; LTV: Lisboa e Vale do Tejo; MSM: men who have sex with men; NA: not applicable; OR: odds ratio.

^a^ OR calculated by univariate logistic regression comparing to baseline. To enable OR calculations, one observation was artificially added to variables with zero-count categories, i.e. MSM, HIV, travel and transmission mode.

^b^ Significance levels: *** p < 0.001, ** p < 0.01, * p < 0.05.

^c^ Confirmed genotyped cases with subgenotype IA, strain VRD 521–2016.

^d^ Baseline period restricted to begin in March 2023, when these variables were introduced in the inquiry.

^e^ Within 15–50 days before symptom onset.

Hepatitis A cases in the outbreak period were more likely to be suspected as sexual transmission by the reporting public health doctors (OR = 18.3; 95% CI: 5.2–88.3). In the outbreak, men constituted 100% of suspected sexual transmission cases, 91% of alimentary/water transmission cases, 50% of close contact cases, and 69% of cases with other/unknown transmission modes.

Since questions regarding HIV and MSM status were only added to the national epidemiologic inquiry in March 2023, we limited the baseline period for these two variables accordingly, to ensure accurate comparisons. Compared with cases in the period March 2023 to Oct 2023, outbreak cases were more likely to be people living with HIV (OR = 8.0; 95% CI: 1.5–148.3) and, among men, more likely to identify as MSM (OR = 19.6; 95% CI: 3.9–358.0). Where data were available, half (11/22) of the outbreak cases reported engaging in group and/or anonymous sex.

Regarding country of birth, outbreak cases were more likely to be born in Brazil compared with baseline cases (OR = 10.8; 95% CI:3.8–38.8). No significant difference was observed in travel history. Cases in this outbreak have generally been mild, with none requiring intensive care or liver transplantation. The high proportion of hospitalised patients (63%) probably reflects the conservative approach adopted by doctors. The education level of current occupation was classified according to the profession reported by the participants, with no significant difference seen between outbreak and baseline cases.

In the sub-analysis, the subgroup of outbreak cases linked by genotyping (IA, strain VRD 521–2016) differed from the baseline period in the same characteristics as in the main analysis. However, due to the smaller sample size, the confidence intervals are wider. To enable OR calculations, one observation was artificially added to variables with zero-count categories, i.e. MSM, HIV, travel and transmission mode (Table).

## Reference laboratory genotyping 

Diagnosis of acute hepatitis A cases was confirmed mainly through serological assays, i.e. the detection of the presence of a specific anti-HAV IgM response. For all confirmed cases, hospitals or private laboratories were asked to forward available specimens (sera, plasma or stools) to the Portuguese National Reference Laboratory Instituto Nacional de Saúde Doutor Ricardo Jorge (INSA) for genotyping. HAV strain characterisation was performed using conventional RT-PCR targeting 460 nt in the VP1/2A junction, according to the HAVNet protocol [6]. Positive amplicons were subjected to Sanger sequencing and sequence analysis was performed to compare with other available reference HAVNet sequences [6] and other international databases.

Molecular typing was performed on 29 of 71 confirmed cases. The result showed that three sequences were 100% identical (VP1–2A region) to the VRD 521–2016 strain, which had cirfculated during a multi-country outbreak among MSM (2016–2018), while 22 sequences were 99.6% identical (458/460 nt) and included the same two nucleotide mutations. The remaining four genotyped samples were identified as two other strains of subgenotype IA, one strain of subgenotype IIIA and one strain of subgenotype IB.

## Early control measures and public health response

The very early detection of this outbreak enabled public health authorities to implement timely control measures and issue an international alert. The primary strategy has been communication campaigns targeting at-risk populations and the general public. In brief, these included general communications via the DGS website, partnerships with local stakeholders such as community-based sexual health centres (Grupo de Ativistas em Tratamentos, GAT) for both digital and traditional outreach, and a push notification to all users of Grindr in Portugal, the largest online dating application used by the LGBTQ+ community. This was enabled by Grindr's social responsibility programme. The GAT also leveraged its Lisbon MSM cohort for an email campaign promoting preventive practices. 

Collectively, these efforts aimed to increase awareness about the infection, symptoms, transmission, hygiene practices and vaccination recommendations. In Portugal, at-risk groups, including MSM, are advised to receive a two-dose hepatitis A vaccination [7], which necessitates a prescription and includes an out-of-pocket expense of ca EUR 20 per dose. In the context of an outbreak, post-exposure vaccination is recommended up to 2 weeks after exposure for contacts who have not been vaccinated or not been fully vaccinated. This was recommended as a part of contact tracing carried out by public health professionals at the local level.

## Discussion

This article describes the early detection and response to a hepatitis A outbreak in Portugal in 2024. Genotyping identified the subgenotype IA VRD 521–2016 strain previously associated with a multi-country MSM-associated outbreak in 2016 to 2018 and the epidemiological investigation reinforced the suspicion of sexual transmission, especially among MSM engaging in group and/or anonymised sex (high-contact MSM). These findings enabled early response measures, directing efforts towards protecting people at risk. At the time of writing, the number of weekly cases has plateaued, although the outbreak is still ongoing.

Compared with the baseline of previous years, hepatitis A cases in the current outbreak were more likely to be young and middle-aged adult men in the Lisbon region, to identify as MSM, to be HIV-positive, and it was more likely that the infection was suspected to be acquired through sexual contact as reported by public health doctors. The lack of noteworthy travel history among cases suggests local transmission rather than infections acquired abroad. Notably, the age–sex profile of outbreak cases, as well as country of birth, resembled that of mpox cases in Portugal (data not shown) and the GAT MSM cohort in Lisbon [8]. This similarity supports the hypothesis of sexual transmission within Portugal, particularly among a subgroup of high-contact MSM.

Genotyping and sequence analysis identified a subgroup within the outbreak that were infected by the same virus strain (VRD 521–2016). This subgroup differed from baseline cases on the same variables as outbreak cases overall (age, sex, region, MSM, HIV status and country of birth) but with higher odds ratios. This suggests that the identified strain circulated predominantly within MSM subgroups. Moreover, one of the cases with the VRD 521–2016 strain was a female suspected to be infected by close contact, which illustrates how the virus may spill over to other groups similar to what has been reported in previous MSM-associated outbreaks [9]. The VRD 521–2016 strain involved in this outbreak has not been identified in any larger outbreaks in Europe since 2018. Interestingly, three cases with VRD 521–2016 strain sequence were identified in the Netherlands in December 2023 [10]. There was no epidemiological link between these cases, but one had reported travelling to Brazil where hepatitis A cases increased in 2023 [11]. 

In the current outbreak in Portugal, the first case linked by genotyping did not report any travel history, thus no clear index case introducing the virus from abroad could be identified. There is a possibility that this virus strain has been circulating unnoticed at low levels in the MSM population since 2018, either in Europe or elsewhere, similar to how hepatitis A virus has been shown to persist among active MSM across Europe for extended periods (years) in the past. [12]. Addressing this hypothesis would require enhanced national and international molecular surveillance efforts for HAV.

Vaccination against hepatitis A is recommended for risk groups in Portugal including MSM, but the vaccine coverage in this group is not well known. In this outbreak, only one case had a record of vaccination in Portugal, but vaccination carried out outside mainland Portugal cannot be excluded. The vaccinated individual was HIV-positive and had received two doses of hepatitis A vaccine. Previous studies have shown waning immunity and a decreased proportion of seroconversion in HIV-positive individuals, especially in patients with low CD4^+^ T cell counts and high HIV RNA load [13]. This suggests that the outbreak occurred due to failure to vaccinate rather than vaccine failure. Possible barriers to vaccination in the affected group may be lack of awareness, out-of-pocket costs and logistical challenges such as prescription requirements.

Public health response measures mainly focused on targeted communication efforts through sexual health clinics and social media, including the dating app Grindr. This encouraged vulnerable individuals to take preventive action to protect themselves from infections, such as getting vaccinated and modifying sexual practices in response to the increased risk, both proven strategies in controlling outbreaks transmitted through sexual contact [14].

Limitations of this investigation include a high degree of missing data on vaccination, the parameter MSM and HIV status, which led to wide confidence intervals. A more accurate recording of vaccination status would enable the exclusion of vaccination coverage as a factor affecting the demographic distribution of cases. Although the Covid-19 pandemic led to a decrease in hepatitis A cases in Europe, this reduction was interestingly not observed in Portugal [4]. Therefore, we consider the baseline period (January 2020 to Oct 2023) to remain relevant for comparison. Furthermore, not all specimens from diagnosed cases were submitted for genotyping, especially the proportion of samples reported as suspected alimentary or water-borne transmission was small. This makes it difficult to determine if some of the cases may be sporadic cases not linked to this particular outbreak. The likely mode of transmission, as assessed by the reporting public health doctors, is subjective, and reporting of this may have been influenced by the heightened attention during the outbreak. This may also have impacted the higher response rate to this question in the outbreak compared with the baseline. Finally, it is worth noting that our surveillance system only identifies symptomatic individuals seeking medical care, thus we are likely to be underestimating the true number of HAV infections.

## Conclusion

Portugal's early detection of this outbreak through its local, regional, and national public health surveillance networks, together with timely genomic analysis, serves as a valuable example for early warning systems. Although the weekly number of cases are not increasing, the outbreak is still ongoing in Portugal. The similarities with outbreaks in the MSM community reported in Europe in recent years highlights the need of increased hepatitis A awareness and vaccination among MSM at higher risk. We hope that our findings here can alert colleagues in other countries to investigate the possibility of hepatitis A spread, especially among high-contact MSM communities.
